# Mirror-Image Analysis of Clinical Treatment and Acceptability in Transitioning from PP1M/PP3M to PP6M in Schizophrenia Outpatients

**DOI:** 10.1192/j.eurpsy.2026.10542

**Published:** 2026-07-31

**Authors:** E. Baca-Garcia, A. M. Serra Segura, P. M. Lopez Rengel, J. Mehawej, J. Antunes, L. Killion

**Affiliations:** 1 Psychiatry, Fundacion Jimenez Diaz; 2 Medical Affairs Neuroscience, Johnson &Johnson Innovative Medicine, Madrid, Spain; 3 Clinical Medical Affairs; 4 Global Medical Affaris Leader; 5 Executive Director EP CDTL CNS, Johnson &Johnson Innovative Medicine, -, United States

## Abstract

**Introduction:**

Paliperidone palmitate 6-month (PP6M), 3-month (PP3M), and 1-month (PP1M) formulations have demonstrated efficacy in improving clinical outcomes and reducing risk of relapse in patients with schizophrenia. Real-world studies have shown that longer dosing intervals are associated with improved clinical outcomes, however, there is minimal evidence comparing the outcomes of these three formulations in clinical practice.

**Objectives:**

This study evaluated the effectiveness of PP6M vs PP3M and PP1M in delaying and preventing relapse among patients with schizophrenia and compared healthcare providers’ treatment satisfaction and healthcare utilization with longer dosing formulations.

**Methods:**

Patient data (n=125) were collected using the MeMind digital ecosystem, covering the period from July 2014 to July 2024 across multiple hospitals in Madrid. Memind registry is representative of the population with schizophrenia that is attended in the participating hospitals. Given the fact that all-registered patients meeting eligibility criteria will be included, sample size estimation is not needed. The number of patients in treatment with PP6M is around 300.Two retrospective treatment phases were considered: one year of prior treatment with PP1M, PP3M, or a combination of both before initiating PP6M, and one year following the initiation of PP6M (1 year mirror image analysis). A Shapiro-Wilk test was performed to assess the normality of the distribution of differences. If the distributions were normal, paired t-tests were conducted to compare the matched cohorts (PP1M vs. PP6M and PP3M vs. PP6M). If the distributions were not normal, a Wilcoxon signed-rank test (nonparametric) was used.

**Results:**

The relapse rate during the PP1M–PP3M period was 9.6% (95% CI: 5.49–16.2), which was almost than double that observed during the PP6M period (4.8%; 95% CI: 1.05–8.55). The hazard ratio for relapse during the PP1M–PP3M period, compared to the PP6M period, was 2.07 (95% CI: 0.78–5.53). 34% of the patients were in monotherapy with PP1M, 49% with PP3M, and 53% with PP6M. The average satisfaction of psychiatrists with paliperidone palmitate long acting injectables was higher than 7.7 out of 10. Fewer visits to psychiatrists were required with PP6M treatment compared to PP1M and PP3M (p<0.001); attendance rates were almost 100%, and visits to mental health nurses’ offices also improved.

**Conclusions:**

Paliperidone palmitate long-acting injectable formulations have shown low relapse rates in real-world settings, with PP6M demonstrating the lowest relapse rate. Combined with high treatment satisfaction and more efficient resource utilization, PP6M may enhance mental health service delivery for patients with schizophrenia.

**Disclosure of Interest:**

E. Baca-Garcia Grant / Research support from: Janssen, Consultant of: Janssen, A. SERRA SEGURA Employee of: Johnson &Johnson, P. LOPEZ RENGEL Employee of: Johnson &Johnson, J. MEHAWEJ Employee of: Johnson &Johnson, J. ANTUNES Employee of: Johnson &Johnson Innovative Medicine, L. KILLION Employee of: Johnson &Johnson Innovative Medicine.

